# Cortisol, leptin and free leptin index (FLI) in newborns in the first days of life and their importance for body weight programming

**DOI:** 10.1186/s13052-019-0743-6

**Published:** 2019-11-09

**Authors:** Beata Kulik-Rechberger, Anna Maria Bury, Anna Rakuś-Kwiatosz, Iwona Beń-Skowronek

**Affiliations:** 1 Department of Paediatric Propedeutics, Medical University of Lublin, ul. A. Gebali 9, 20-091 Lublin, Poland; 2 Department of Paediatric Endocrinology and Diabetology, Medical University of Lublin, Lublin, Poland

**Keywords:** Newborn, Cortisol, Leptin, Soluble leptin receptor

## Abstract

**Background:**

Birth weight and leptin seem to be the factors responsible for early programming of body weight in later life. A marker for leptin action is free leptin index (FLI), which depends on soluble leptin receptor (Ob-Re) (FLI = leptin/Ob-Re). In the present article, we suggest that FLI is modulated partly by cortisol variations observed in newborns in the first days of life and is connected with their postnatal weight loss.

**Methods:**

The study group consisted of 44 full-term newborns. Leptin, cortisol and Ob-Re concentrations were determined in the umbilical cord blood (UCB) and in the newborns’ blood (NB) on the fourth day of life, free leptin index (FLI = leptin/Ob-Re) was calculated. Correlations between the assessed parameters and the somatic features of the newborns were examined.

**Results:**

Birth weight, length and chest circumference of newborns were positively correlated with leptin concentration in the UCB but not with FLI in the UCB. Cortisol and leptin concentrations, as well as FLI values declined concomitantly with body weight, and were lower on the fourth day of life than on the first one; however, Ob-Re concentration increased (*p* < 0.0001). There was a positive correlation between the newborns’ birth weight loss percentage evaluated on the fourth day of life and FLI in newborns (*R* = 0.39; *p* < 0.01). Positive correlations between cortisol and Ob-Re in UCB (*R* = 0.35; *p* < 0.02) and in NB (*R* = 0.36; *p* < 0.01), as well as a negative correlation between cortisol and FLI (*R* = -0.32; *p* < 0.03) in NB were noted.

**Conclusions:**

Our data suggest a possible relationship between cortisol and a soluble leptin receptor (Ob-Re), which changes free leptin index (FLI) and is connected with birth weight loss in newborns. Whether these observations are important for programming of future body weight of children requires further research.

## Background

Both foetal and neonatal periods are critical for overall human development [1]. The processes of growth and maturation during this time include the development of the central nervous system, the formation of the trajectories of circuits, as well as the programming of their functions [1]. Such programming also provides grounds for further functional development of the body at the cell, tissue, and organ levels, and may have further consequences for appetite regulation and the manifestation of obesity [1]. Leptin seems to be a key hormone responsible for such programming [2]. It is involved in the process of hypothalamus maturation and in the regulation of hypothalamic-pituitary axis activity, which results in the production of growth hormone, thyroid hormones, and glucocorticoids [2]. Studies in animal models demonstrate that the focus of developmental programming lies within the adipose tissue; research has revealed that the adipose tissue of the offspring from malnourished dams had undergone modifications resulting in the reprogramming of its metabolism. Indeed, during the perinatal period, the sensitivity of the adipose tissue can be modified by certain hormone levels [3]. High levels of leptin and leptin receptor expression in maternal tissues (particularly in the third trimester of pregnancy), as well as in the placenta and the foetus suggest that leptin may be an important factor in foetal growth and development [4–6]. In rodents, for example, in the first few weeks after birth, high levels of leptin parallel the development of the hypothalamus and nerve cell junctions involved in food intake [7].

Leptin activity is modulated by the soluble leptin receptor (Ob-Re), which is the main leptin binding protein in the blood [8]. It renders leptin temporarily inactive, and, in the cells of different tissues, it modulates leptin uptake. Moreover, it protects leptin from degradation and elimination from the body [9]. The level of Ob-Re can provide an indication of free leptin. This is assessed by the free leptin index (FLI), i.e., the ratio of leptin to Ob-Re [10].

One of the factors that presumably contribute to programming the body weight of children and adults is their birth weight, as energy supply disorders in the fetus or neonate result in lifelong programming of the individual’s body weight set point [3]. Another one is cortisol, which modulates the regulation of the genes involved in growth and maturation. The fetal/neonatal hypothalamus-pituitary-adrenal axis is prone to long-term programming by glicocorticosteroids, and, presumably, their effects may persist throughout the life [11].

The aim of the present study was to determine the relationships between cortisol, Ob-Re, leptin, and FLI in newborns, in the first days of life. This could help gain a better understanding of the critical metabolic changes occuring in this short period.

## Methods

The study group consisted of 44 healthy neonates, hospitalized at St. John’s Hospital in Lublin (Poland). The neonates were full-term (≥38 weeks), naturally born, appropriate for gestational age, with an Apgar score ≥ 7 at 1 min, from single and uncomplicated pregnancies. Due to the newborns’ need to adapt to extrauterine life, the first anthropometric measurements (here referred to as birth measurements) were taken between the second and fourth hour after birth. All the newborns stayed with their mothers (the rooming-in system) and were breastfed. The body weight of the newborns was measured over the course of four consecutive days. In each newborn, the process of adaptation to extrauterine life was uneventful, and the weight loss, as evaluated on the fourth day of life, did not exceed 10% of birth weight. Table 1 presents the characteristics of the examined group of children.

**Table 1:** Clinical characteristics of the study population

Study population (*n* = 44)	
Gender (male)	27 (61,4%)
Gestational age (wks)	39.5 ± 1.1
Apgar score (range)	8–10
Birth weight (g)	3432 ± 409
Weight on the 2nd day (g)	3298 ± 401
Weight on the 3rd day (g)	3275 ± 408
Weight on the 4th day (g)	3340 ± 410
Birth weight loss on the 4th day (%)	5.47 ± 2.45
Body length (cm)	53.9 ± 2.5
Head circumference (cm)	34.3 ± 1.3
Chest circumference (cm)	33.5 ± 1.5
Exclusive breastfeeding	44 (100,0%)

Data expressed as mean ± SD, range or n (%)

The blood for biochemical scores was drawn from the umbilical cord (mixed venous and arterial blood) and from the peripheral vein of newborns on the fourth day of life. The sera were stored at –20 °C until analysis. Leptin, Ob-Re and cortisol concentrations were measured in the serum of the umbilical cord blood and the newborns’ blood. Leptin concentration was assessed by commercially available radioimmunoassay, using the Human Leptin RIA Kit (Linco Research Inc., St. Charles, USA), while Ob-Re concentration was identified via the Human Leptin Soluble Receptor ELISA Kit (Diagnostic System Laboratories, Inc., UK). Leptin and Ob-Re concentrations were used to calculate the free leptin index (FLI) according to the following formula: FLI = leptin (ng/ml) / Ob-Re (ng/ml). Cortisol concentration was measured by radioimmunoassay, using the Cortisol RIA test (Immunotech, The Czech Republic).

The results were analysed statistically, with the use of Wilcoxon Signed Rank Test. Correlations between two measurable parameters were examined by Spearman’s rank correlation coefficient R test; herein, 5% deduction error and *p* < 0.05 were assumed statistically significant. For all the parameters and with the size of the examined group, the power of the test was above 0.95, at alpha = 0.05. Statistical analyses were performed with the use of STATISTICA v.8.0 (StatSoft, Poland) computer software.

All the mothers signed informed consent forms to participate in the study. The research was approved by the Bioethical Commission of the Medical University of Lublin.

## Results

As was expected, the average body weight of the newborns on the fourth day of life was considerably lower than their birth weight (Table 1). In the comparison of serum leptin concentration, it was nearly three times higher in the umbilical cord blood than in the newborns’ blood on the fourth day of life (*p* < 0.0001) (Table 2). The analysis of Ob-Re concentration found that it was much higher in the newborns’ blood on the fourth day of life than in the umbilical cord blood (*p* < 0.0001). The value of FLI in the newborns’ peripheral blood was lower than in the umbilical cord blood (*p* < 0.0001). Cortisol concentration decreased as well and was nearly three times lower in the newborns’ blood on the fourth day of life (*p* < 0.0001) than in the umbilical cord blood (Table 2). There was a positive correlation between serum leptin concentration in the umbilical cord blood and birth weight (*R* = 0.35; *p* < 0.03), body length (*R* = 0.24; *p* < 0.001), and chest circumference (*R* = 0.30; *p* < 0.004) on the first day of the newborns’ life (Table 3). There was also a positive correlation between leptin concentration in the newborns’ blood on the fourth day of life and their body weight on the same day (*R* = 0.38; *p* < 0.001) (Table 3). Correlations between FLI in the umbilical cord blood and the newborns’ somatic features were not found. The analysis of the percentage of newborns’ birth weight loss revealed that it was negatively correlated with Ob-Re concentration (*R* = -0.29; *p* < 0.05) and positively correlated with the FLI value (*R* = 0.39; *p* < 0.01) evaluated on the fourth day of life (Table 3).

**Table 2:** Leptin, soluble leptin receptor (Ob-Re), cortisol concentrations and free leptin index (FLI) in the umbilical cord and in newborns on the 4th day of life

Parameter	Umbilical cord blood	Newborns’ blood	*p*-value
Leptin (ng/ml)	10.37 ± 6.35	3.44 ± 1.30	*p* < 0.0001
Ob-Re (ng/ml)	4.44 ± 3.11	15.00 ± 5.67	*p* < 0.0001
FLI	2.91 ± 2.69	0.26 ± 0.14	*p* < 0.0001
Cortisol (nmol/L)	325.01 ± 226.45	107.26 ± 81.35	*p* < 0.0001

Data expressed as mean ± SD

*FLI* = Leptin/Ob-Re

**Table 3:** Correlations between leptin, soluble leptin receptor (Ob-Re), free leptin index (FLI) and cortisol concentrations and the somatic features of the newborns

Parameter	Somatic features of the newborns					
	1st day of life				4th day of life	
	birth weight	body length	head circumference	chest circumference	weight	birth weight loss (%)
Leptin in UCB	*R* = 0.35 *p* < 0.03	*R* = 0.24 *p* < 0.001	ns	*R* = 0.30 *p* < 0.004	ns	ns
Ob-Re in UCB	ns	ns	ns	ns	ns	ns
FLI in UCB	ns	ns	ns	ns	ns	ns
Cortisol in UCB	ns	ns	ns	ns	ns	ns
Leptin in NB	–	–	–	–	*R* = 0.38 *p* < 0.001	ns
Ob-Re in NB	–	–	–	–	ns	*R* = -0.29 *p* < 0.05
FLI in NB	–	–	–	–	*R* = 0.31 *p* < 0.04	*R* = 0.39 *p* < 0.01
Cortisol in NB	–	–	–	–	ns	ns

*UCB* the umbilical cord blood, *NB* newborns’ blood, *FLI* Leptin/Ob-Re, *ns* statistically not significant, *R* Spearman’s rank correlation coefficient, - not considered

The analysis of relations between the investigated parameters (Table 4) showed statistically significant positive correlations between the concentrations of cortisol and Ob-Re in the umbilical cord blood (*R* = 0.35; *p* < 0.02) and in the newborn’s blood on the fourth day of life (*R* = 0.36; *p* < 0.01), while a negative correlation was seen between cortisol concentrations and the value of FLI in the newborns’ blood (*R* = -0.32; *p* < 0.03) (Table 4).

**Table 4:** Correlations between leptin, soluble leptin receptor (Ob-Re) and free leptin index (FLI) and cortisol concentration in umbilical cord blood (UCB) and in the newborns blood (NB)

Parameter	Cortisol		Leptin	
	UCB	NB	UCB	NB
Leptin in UCB	ns	ns	–	ns
Leptin in NB	ns	ns	ns	–
Ob-Re in UCB	R = 0.35 *p* < 0.02	ns	ns	ns
Ob-Re in NB	ns	R = 0.36 *p* < 0.01	ns	ns
FLI in UCB	ns	ns	*R* = 0.69 *p* < 0.001	ns
FLI in NB	ns	R = -0.32 *p* < 0.03	ns	*R* = 0.59 *p* < 0.001

*UCB* umbilical cord blood, *NB* newborns’ blood, *FLI* free leptin index, *ns* statistically not significant, *R* Spearman’s rank correlation coefficient, - not considered

## Discussion

Accumulated evidence reveals the long-term consequences of birth weight and nutrition in early life. Indeed, nutritional status and the level of numerous actively functioning proteins (including leptin) in the period of intrauterine and early extrauterine life may affect the development of hypothalamic structures involved in appetite control, energy expenditure and metabolism, as well as adipose tissue programming by epigenetic mechanisms [1–3]. Similarly to other authors, we found that there was a positive correlation between leptin concentration in the umbilical cord blood and the newborns’ somatic features [12–17]. The decrease of leptin concentration after birth was accompanied by the loss of newborn’s body weight, as recorded until the fourth day of life. This could be observed due to the fact that the placenta is a leptin source in the foetal circulation [18], but such variations can also occur due to metabolic changes in the newborn during the process of adaptation to extrauterine life.

Bellone et al. found that leptin levels in the umbilical cord blood correlated positively with newborn’s birth weight, but on the fifth day, the association between cord blood leptin and weight was lost [19]. In our study, the leptin concentration in the umbilical cord blood correlated positively with newborns’ weight on the first day of life but not on the fourth day. Similarly to the results of Valūniene et al. [20], we did not find correlation between leptin level in the umbilical cord blood and postnatal weight change of the neonates. Chaoimh and co-authors [21] published the data illustrating associations between higher cord blood leptin and slower weight gain of newborns between birth and 2 months of age. However, Fonseca et al. put forward that high levels of leptin in the umbilical cord blood predicted higher birth weight and lower weight loss in the immediate postnatal period [22]. Moreover, Wang et al. noted that the serum leptin levels were significantly decreased and positively correlated with the neonates’ body weight gain in the first week of life [23]. Similarly to our study, they found a positive correlation between leptin concentration and newborns’ body weight on the fourth day of life. Based on the results of their study, they concluded that a rapid decline in serum leptin after birth is associated with greater future weight gain and a physiological advantage for infants. Our results suggest that the rapid decrease in leptin levels after birth could be mediated by hormonal changes, and that cortisol can play a part in this [24].

Furthermore, Street and co-authors established that placental cortisol concentration correlated positively with weight gain during the first 5 years of postnatal growth [25]. In our neonates, cortisol concentration decreased with time, and, on the fourth day, was lower than that in the umbilical cord blood. However, we did not find any correlations between leptin and cortisol concentrations, either in the umbilical cord blood or in the peripheral blood. This result is similar to that of other authors [26, 27] and could imply a possible relationship not between cortisol and total leptin, but with its active form. We also did not find any correlation between cortisol and the body weight change. Of note, we did find positive correlations between cortisol and Ob-Re concentrations, and, on the fourth day, neonates’ Ob-Re concentration was higher than that in the umbilical cord blood. The work of Kratzsch et al. [13] also showed decreased leptin and increased Ob-Re concentration in newborns during the first week of life. Moreover, they found that leptin concentration in the umbilical cord blood correlated positively with birth weight on the first, third, and fifth days of life, while Ob-Re/leptin quotient correlated negatively. In contrast, Marino-Ortega et al., similarly to our study, did not find any correlation between cord blood soluble leptin receptor levels and the neonates’ somatic parameters [28]. We also did not find correlations between leptin and Ob-Re concentration. This result is in agreement with that of other investigators [13].

It can be presumed that decreased leptin (particularly, free leptin) concentration stimulates an infant to suck, and, as a result, enhances the amount of available nutritional elements, which, in turn, positively determines the growth rate. This effect is achieved via the mechanism triggered by starvation, when a low level of leptin decreases ‘the leptin satiety signal’. The decrease of leptin concentration may also be necessary to reduce energy expenditure and maintain proper body temperature [29, 30]. Adaptation mechanisms already seen in the foetal period are associated with the development of the central nervous system. An experimental study showed that leptin participates in this process. Herein, the hormone crosses the blood-brain barrier by interacting with its receptor. Pan et al. discovered that in mice, the expression of the leptin receptor in cerebral microvessels undergoes developmental changes [30]. Furthermore, the authors revealed that higher expression of Ob-Ra, Ob-Rb, Ob-Rc, and Ob-Re isoforms of the leptin receptor is seen in baby-mice than in adults. This seems to ensure easy leptin access to the structures being created. However, a relatively high expression of Ob-Re impedes the hormone transport to the brain, which promotes food ingestion.

Our study suggests that Ob-Re concentration in the blood of newborns can be modulated by cortisol. We found a positive correlation between cortisol and Ob-Re concentration in the umbilical cord blood and in the blood of newborns on the fourth day of life, as well as a negative correlation between cortisol and FLI on the fourth day of life. The findings may also suggest that cortisol modulates leptin activity by influencing its receptor concentration. In addition, it was noted that birth stress and increase of cortisol level stimulate metalloproteinase activity, which results in increased Ob-Re concentration [31], and higher Ob-Re results in lower free leptin. Free leptin is able to cross the blood-brain barrier and affect appetite and energy expenditure. Contrary to the study results of Papageorgiou et al., we did not find any correlation between cortisol and leptin concentration [32]. Moreover, some authors suggest that, in conditions of insufficient food availability, there is a specific central resistance to leptin which may occur in a neonate over the first days after birth [33]. Decrease of leptin level and sensitivity to leptin not only seems to protect a newborn from excessive birth weight loss, but also seems to determine food intake in the period when mechanisms controlling this process have not yet been fully developed. However, a limitation to our study is the lack of a follow-up observation. A recent study on leptin as a potential modulator of developmental programming of childhood obesity indicates that children with high cord blood leptin (>90th percentile) exhibit lower weight, height, and body mass index from 6 months to early childhood [34]. Moreover, increased cord blood leptin is weakly associated with increased fat mass in late childhood but is not associated with it in adolescence [35]. These studies seem to confirm that leptin prevents obesity. Our research draws attention to changes in leptin concentration immediately after birth, as well as changes in its free fraction, which is biologically more active. Free fraction of leptin seems to depend on the cortisol concentration. It is possible that the changes observed in the concentration of leptin, its free fraction, and of cortisol immediately after birth affect the programming of the child’s appetite and future body weight.

## Conclusions

Our data suggest a possible relationship between cortisol and soluble leptin receptor (Ob-Re), which changes free leptin index (FLI) and is connected with birth weight loss in newborns. Whether these observations are important for programming children’s future body weight requires further research.

## Data Availability

The datasets used and/or analysed during the current study are available from the corresponding author on reasonable request.

## Abbreviations

FLI: Free leptin index
NB: Newborns’ blood
Ob-Re: Soluble leptin receptor
UCB: Umbilical cord blood
